# Shugan Decoction Alleviates Colonic Dysmotility in Female SERT-Knockout Rats by Decreasing M_3_ Receptor Expression

**DOI:** 10.3389/fphar.2020.01082

**Published:** 2020-09-11

**Authors:** Yinshu Wang, Ying Dong, Enkang Wang, Yangyang Meng, Zijuan Bi, Shuai Sun, Chaochao Zhang, Haiting Fan, Jianye Yuan

**Affiliations:** ^1^ Institute of Digestive Diseases, Longhua Hospital, Shanghai University of Traditional Chinese Medicine, Shanghai, China; ^2^ School of Public Health, Shanghai University of Traditional Chinese Medicine, Shanghai, China; ^3^ Institute of Chinese Materia Medica, Shanghai University of Traditional Chinese Medicine, Shanghai, China; ^4^ Laboratory Animal Center, Shanghai University of Traditional Chinese Medicine, Shanghai, China

**Keywords:** serotonin reuptake transporter, Shugan decoction, intestinal motility, muscarinic receptor, irritable bowel syndrome, rats

## Abstract

**Background:**

Irritable bowel syndrome (IBS) is a functional gut disease characterized by visceral hypersensitivity and gut motor dysfunction. Serotonin (5-hydroxytryptamine, 5-HT) is an important enteric neurotransmitter. High levels of 5-HT aggravate IBS symptoms. The serotonin reuptake transporter (SERT) is a membrane-embedded transporter involved in IBS pathogenesis that plays an important role in regulating 5-HT signaling.

**Aim:**

We investigated whether gut motor function was altered in SERT-knockout (SERT-KO) rats. Additionally, we sought to determine whether Shugan decoction (SGD), a clinically experienced prescription for the treatment of IBS, exerts regulatory effects on intestinal motility in SERT-KO rats, and attempted to identify the mechanisms involved.

**Method:**

SERT-KO rats were produced by transcription activator-like effector nuclease (TALEN) technology. Fecal pellet output was measured for ten consecutive days to estimate distal colonic motility. Small intestinal motility was measured by charcoal-meal experiments. The colonic and small intestinal muscle contractile activities were measured by organ bath study. Western blot was used to analyze the muscarinic receptor expression in colon tissue.

**Result:**

Compared with that in wild-type (WT) rats, the defecation amount, amplitude of spontaneous contraction, and the tension of ACh-induced contraction of colonic longitudinal smooth muscle in SERT-KO rats were significantly increased. The expression of muscarinic receptor subtype-3 (M_3_R) in the colons of SERT-KO rats was also elevated. SGD can decrease defecation of SERT-KO rats. Moreover, SGD reduced the amplitude of spontaneous contraction, the frequency and tension of ACh-induced contraction of colonic longitudinal smooth muscle, and the expression of M_3_R in the colon in SERT-KO rats.

**Conclusions:**

SERT-KO rats showed increased defecation accompanied by enhanced colonic motility and M_3_R expression. The findings suggest that SGD modifies colonic dysmotility and reduces defecation in SERT-KO rats by down-regulating M_3_R expression in the colon.

## Introduction

Irritable bowel syndrome (IBS) is a chronic functional bowel disorder characterized by abdominal pain, with altered bowel habits and abdominal distention and bloating (Sultan and Malhotra, 2017). According to epidemiological research, the incidence of IBS is about 11.2% worldwide (Lovell and Ford, 2012) and 4.6% in China (Enck et al., 2016), with a higher prevalence in women than in men (Gwee et al., 2018). The incidence of IBS increases every year. Although there are no obvious structural or biochemical abnormalities underlying the development of IBS and it is not a life-threatening condition, the frequency and unpleasantness of the symptoms have a severe negative impact on patients’ quality of life.

According to the latest diagnostic criteria, Roman IV, IBS can be classified into four subtypes: constipation-predominant IBS (IBS-C), diarrhea-predominant IBS (IBS-D), mixed IBS (IBS-M), and unclassified IBS (IBS-U) (Drossman, 2016). At present, although the pathogenesis of IBS is not fully understood, it is considered to be linked to changes in intestinal flora, visceral sensory abnormalities, gastrointestinal motility abnormalities, brain-intestinal axis changes, intestinal mucosal immune activation, and increased intestinal permeability (Crisan and Dumitrascu, 2014); among these, visceral hypersensitivity and gastrointestinal motility abnormalities represent as the main pathophysiological factors.

Recent advances in research techniques and the consequent in-depth understanding of gastrointestinal motility has led to IBS being recognized as a gastrointestinal motility disorder. However, the exact relationship between the specific patterns of changes in gastrointestinal motility and IBS symptoms is still unclear, and the mechanism involved is poorly understood. Gut motility can be modulated by a variety of factors including food intake, physical activity levels, the autonomic nervous system, gastrointestinal hormones, and emotional and psychological factors (Burns, 1980). Gastrointestinal hormones and neurotransmitters play different roles under specific conditions.

5-hydroxytryptamine (5-HT, also known as serotonin or enteramine) is an important neurotransmitter and signaling molecule that regulates gastrointestinal motility, sensation, and secretion. Studies have shown that circulating 5-HT levels in IBS-D patients are significantly higher than in healthy individuals, suggesting that 5-HT may be related to IBS-D onset (Mawe and Hoffman, 2013). Furthermore, 90-95% of 5-HT is released from intestinal chromaffin cells and inactivated by the serotonin reuptake transporter (SERT) in the membrane. Through uptake of 5-HT, SERT regulates the amount of extracellular 5-HT and the duration of its interaction with 5-HT receptors to control signaling transmission. Studies have shown that *SERT* expression is closely related to IBS, and certain *SERT* genetic polymorphisms may increase individual susceptibility to IBS (Jarrett et al., 2007).

In addition to gut-related symptoms, IBS patients suffer from comorbidities such as depression and anxiety, similar to the clinical manifestation of incoordination between the liver and the spleen as postulated in traditional Chinese medicine (TCM). According to TCM, treatment for this condition mainly involves regulating liver and spleen function with Chinese medicines. Shugan decoction (SGD) is a Chinese medicine formula that has been reported to be effective in treating IBS-D patients with incoordination between the liver and the spleen (Shang et al., 2013; Lu et al., 2018).

The present study aims to investigate the changes in intestinal motility in IBS. To this end, we knocked out the *SERT* gene in rats, and examined whether the resulting SERT-knockout (SERT-KO) rats showed intestinal abnormalities similar to IBS. Then, we investigated the effect of SGD in regulating intestinal motility and the potential underlying mechanism to explore its potential for clinical application.

## Materials and Methods

### Agents and Materials

*White attractylodes rhizome* (*Baizhu*) (Shang Hai De Hua GuoYao; Lot number: 2018061101), *white peony root* (*Baishao*) (Shang Hai Hua Pu Zhong Yao; Lot number: 2018042901), *dried old orange peel* (*Chenpi*) (Shang Hai Lei Yun Shang Zhong Yao; Lot number: 1805037), *ledebouriella root* (*Fangfeng*) (Shang Hai Yu Tian Cheng Zhong Yao; Lot number:2017022706), and *Radix bupleuri* (*Chaihu*) (Ma Chen Jiu Zhou; Lot number: E2018050101), which form the components of SGD, were purchased as crude herbs from the JinKe Pharmacy (Shanghai, China). Saikosaponin A (National Institute for Food and Drug Control; Lot number: 110777-201912), paeoniflorin (National Institute for Food and Drug Control; Lot number: 110736-201943), 5-O-Methylvisammioside (National Institute for Food and Drug Control; Lot number: 111523-201811), hesperidin (National Institute for Food and Drug Control; Lot number: 110721-201818), and cimicifugoside (National Institute for Food and Drug Control; Lot number: 111522-201913) were purchased from the Shanghai Zhaorui Biological Technology Co. (Shanghai, China). Muscarinic receptor subtype-2 (M_2_R) antibody (Abcam: ab109266); muscarinic receptor subtype-3 (M_3_R) antibody (Abcam: ab87199); HRP secondary antibody (CWBIO: 111-035-003); β actin antibody (CWBIO: ET1702-67); and acetylcholine (Sigma: C4382-10G) were additionally obtained.

### Preparation of SGD Extract

SGD consists of Radix bupleuri (Chaihu), dried old orange peel (Chenpi), white attractylodes rhizome (Baizhu), white peony root (Baishao), and ledebouriella root (Fangfeng) in a mass ratio of 6:3:6:4:4. Water extract of SGD was prepared by the Herbal Chemistry Lab in Shanghai University of TCM. The procedure used was as follows: herbs were soaked in water 6 times for 1 h and then boiled for 1 h. Next, the mixture was filtrated with 4 layers of gauze, and the filtrate was collected. The above procedure was repeated twice, and the filtrate was dried to obtain powder.

### Analysis of the Chemical Composition of SGD by Chromatography

#### Preparation of SGD Sample Solution

The SGD extract (500 mg) was weighed and dissolved in purified water to shake thoroughly using a sonicator for 40 min. The volume of this solution was set to 40 mL. Then, 1 mL was transferred to a C_18_ column activated by methanol. The column was eluted successively with 10 mL of purified water and 10 mL of methanol. The methanol eluent was collected and dried under reduced pressure. The residue was then dissolved with 1 mL of methanol and passed through a microporous membrane (0.45 μm) and the subsequent filtrate was collected as an SGD sample solution with a concentration of 49.80 mg/mL.

#### Preparation of Reference Substance Solutions (RSS)

Five milligrams of saikosaponin A, paeoniflorin, 5-O-methylvisammioside, hesperidin, and cimicifugoside were weighed and dissolved with an appropriate amount of methanol to obtain a final concentration of 1.0 mg/mL.

### High-Performance Liquid Chromatography (HPLC) Analysis

The samples of SGD solution and RSS were analyzed using a Dionex UltiMate™ 3000 RSLC nano system (Thermo Scientific, MA, USA) equipped with a Corona^®^ ultra™ CAD detector, Luna^®^ C18 Column (Phenomenex, 250×4.6 mm, 5 μm), and a data station with analytical software (CHROMELEON^®^). Mobile phases consisted of A-Purified water and B-acetonitrile. Gradient was set as follows: 0 min, 5% B; 35 min 65.5% B; 35.001 min, 100% B; 40 min, 100% B. Column temperature was set at 25°C and the nebulization temperature of CAD was high.

### Animals and Treatments

Three male and three female SERT-KO rats were produced, using transcription activator-like effector nuclease (TALEN) technology, by Cyagen Biosciences Inc., Suzhou, China. After timely propagation, 12 female SERT-KO rats with the homozygous *SERT* mutation, as confirmed by gene sequencing, were used in this study. In addition, 6 wild-type (WT) female Sprague-Dawley (SD) rats were purchased from the Shanghai Slac Laboratory Animal Co. Ltd. All animals weighed 200 ± 20 g and were housed at the experimental animal center of Shanghai University of TCM, under a 12/12 light cycle, at standard temperature (21-24°C) and humidity (50% ± 5%), and with *ad libitum* access to standard rat chow and tap water. SERT-KO rats were randomly divided into two groups, with 6 rats in each group. One group was gavaged with SGD (1.28 g/kg body weight, crude drug dose, once per day) for 7 consecutive days. The second group and the WT rats were gavaged with the same volume of saline. All the animal experiments were approved by the Animal Ethics Committee of Shanghai University of TCM (No. PZSHUTCM191206008) and conducted between 8:00-11:00 AM to minimize potential confounding effects of diurnal variations.

### Rat Defecation

To assess distal colonic motility, fecal pellet output in the second hour after the rats were gavaged with SGD or saline) was counted every day, for 7 consecutive days.

### Small Intestinal Transit

The animals were orally gavaged with activated carbon in double-distilled water after fasting for 24 h. The whole intestine was dissected and was stretched along a line naturally. The distance of activated carbon transit and the full length of the small intestine (from pylorus to ileocecal valves) were measured with a ruler. Small intestinal transit rate (%) was assessed by calculating the percentage ratio of activated carbon transit over the full small intestinal length.

### Organ Bath Study

The rats were euthanized with chloral hydrate. The ileum and distal colon tissue were removed and were placed in ice-cold Krebs solution (mM): NaCl 130, NaHCO_3_:14.4, MgCl_2_·1.2, KCl 6, NaH_2_PO_4_ 1.2, CaCl_2_ 0.277, and glucose 2.18, which was bubbled with 95% O_2_–5%CO_2_ at a pH of 7.4. Following careful removal of the mucosa and submucosa by sharp dissection, smooth muscle strips (approximately 0.8 cm length and 0.2 cm width) were obtained. The longitudinal smooth muscle strips were mounted between two stainless steel hooks vertically in an organ bath filled with 10 ml of Krebs solution maintained at 37°C and bubbled with a mixture of 95% O_2_–5% CO_2_. The upper hook connected to an isometric force transducer [TRI201AD, Cornella (BCN), Spain). A resting tension of 0.5 g was slowly applied to the muscle strips. The muscle strips were allowed to equilibrate for 60 min. Then, they were randomly stimulated by ACh (10^-4^ mol/L) or KCl (30 mmol/L) for 30 min. The spontaneous and ACh- or KCl-induced tension (g), frequency (cycle/min), and amplitude (g) were measured. To measure the small intestinal muscle contractile activity, analysis was performed for one-minute periods before and after each drug was added to the organ bath. Additionally, the colonic muscle contractile activities were recorded for five minute-periods before and after the drugs were added. The data for the contractile activity of the muscle strips were amplified using the Octal bridge amplifier (AD Instruments, Castlehill, Australia) and processed using PowerLab (8/35, AD Instruments, Castlehill, Australia).

### Western Blot

For protein analysis, the ileum and colon tissue were cut into small pieces. Total proteins were extracted using RIPA lysis buffer and subjected to centrifugation at 12000 rpm, 4°C for 15 min. Supernatants were collected and protein concentration was determined using the BCA protein assay kit (CWBIO: CW0014s). Samples were mixed with 5× loading buffer and heated at 95°C for 5 min to denature the proteins. Then, 80 mg of total proteins were loaded on 10% SDS polyacrylamide gels and electrophoresed. The separated proteins were transferred to PVDF membranes (Millipore, Darmstadt, Germany), and the membranes were incubated in 5% skimmed milk at room temperature for 1 h to block nonspecific binding. The membranes were then incubated overnight at 4°C with the M_2_R antibody (1:1000), M_3_R antibody (1:2000), or the β-actin antibody (1:1000). After washing three times with tris-buffered saline and Tween 20 (TBST) for 10 min, incubation was performed with the corresponding secondary antibody (Sheep anti rabbit-HRP secondary antibody 1:1000) conjugated to horseradish peroxidase for 1 h at room temperature, followed by three washes in TBST for 10 min. Specific protein bands were visualized using the ECL kit (Millipore: WBKLS500) and imaged with SYNGENE (G: BOX Chemi XT4).

### Statistical Analysis

SPSS version 21.0 (SPSS, Chicago, IL, USA) and GraphPad Prism 5 (La Jolla, CA, USA) were used for data analysis. Each value was expressed as mean ± SE. Diﬀerences between groups were analyzed by one-way analysis of variance (*ANOVA*) followed by Dunnett’s test. *P* < 0.05 was considered statistically signiﬁcant.

## Results

### Chemical Composition of SGD

**Figure 1:**
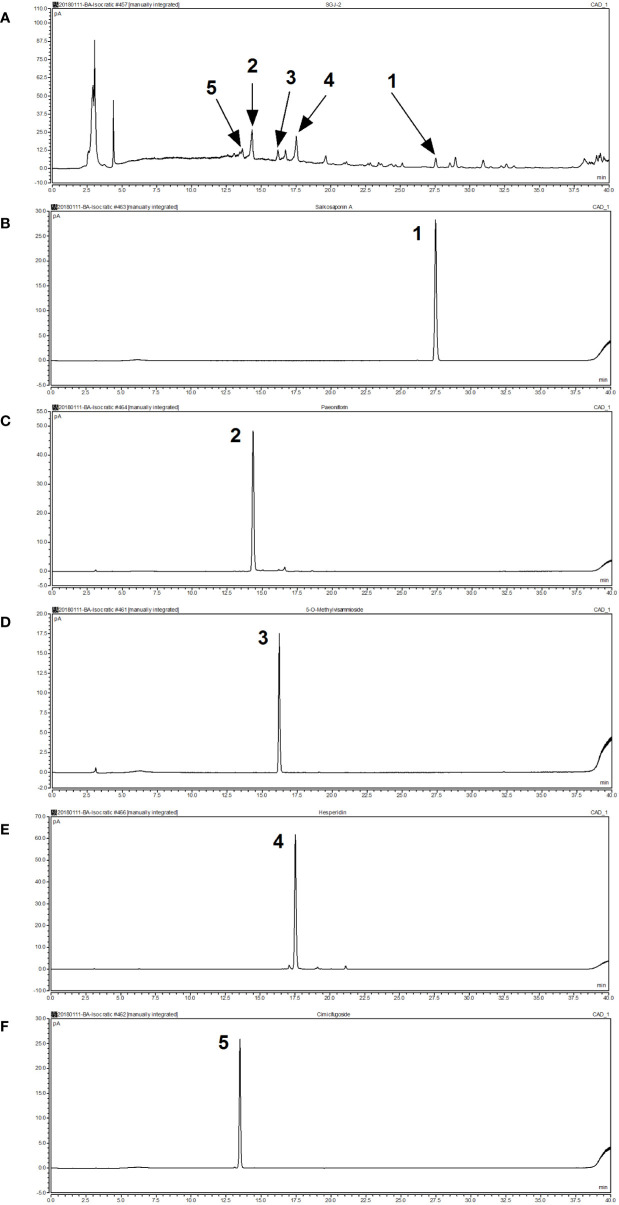
Analysis of the chemical composition of Shugan decoction (SGD) extract by HPLC. **(A)** HPLC chromatogram of SGD extract **(B)** HPLC chromatogram of saikosaponin A **(C)** HPLC chromatogram of paeoniflorin **(D)** HPLC chromatogram of 5-O-methylvisammioside **(E)** HPLC chromatogram of hesperidin **(F)** HPLC chromatogram of cimicifugoside. Peak 1: saikosaponin A; Peak 2: paeoniflorin; Peak 3: 5-O-methylvisammioside; Peak 4: hesperidin; Peak 5: cimicifugoside.

We confirmed the principal ingredients of the SGD extract, on the basis of its herbal composition according to the Chinese Pharmacopeia, to be as follows: saikosaponinA, paeoniflorin, 5-O-Methylvisammioside, hesperidin, and cimicifugoside (**Figure 1**).

### Defecation of SERT-KO Rats and of the Effects of SGD

**Figure 2:**
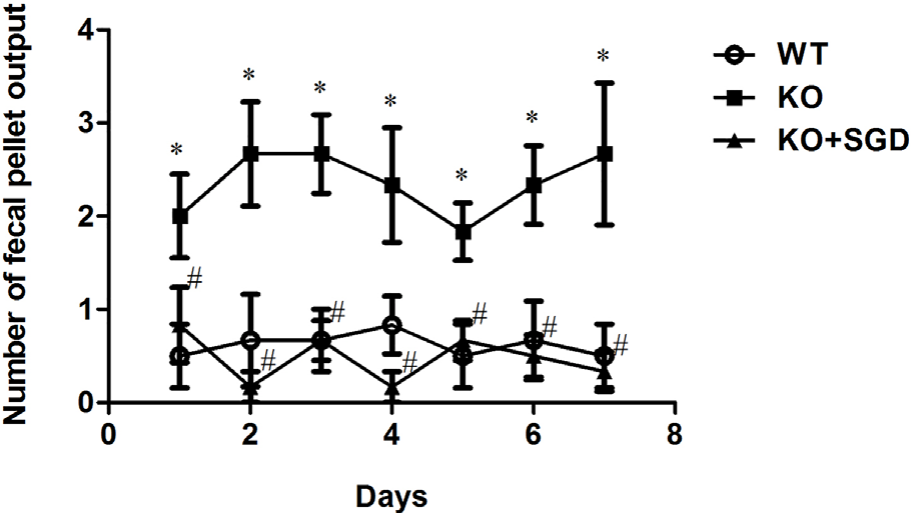
Fecal pellet numbers produced by rats every day and the effect of SGD. All results expressed as mean ± SE (n = 6/group, *P < 0.05 *vs.* WT, ^#^ *P* < 0.05 *vs.* KO).

SERT-KO rats produced significantly larger numbers of fecal pellets than WT rats (*P* < 0.05), indicating that SERT-KO resulted in increased defecation in rats. From the second day post-SGD intervention onward, the defecation of SERT-KO rats gavaged with SGD was significantly reduced compared with that in SERT-KO rats fed with saline (*P* < 0.05), indicating that SGD improved the defecatory status of the SERT-KO rats (**Figure 2**).

### Small Intestinal Propulsion in SERT-KO Rats and the Effects of SGD

**Figure 3:**
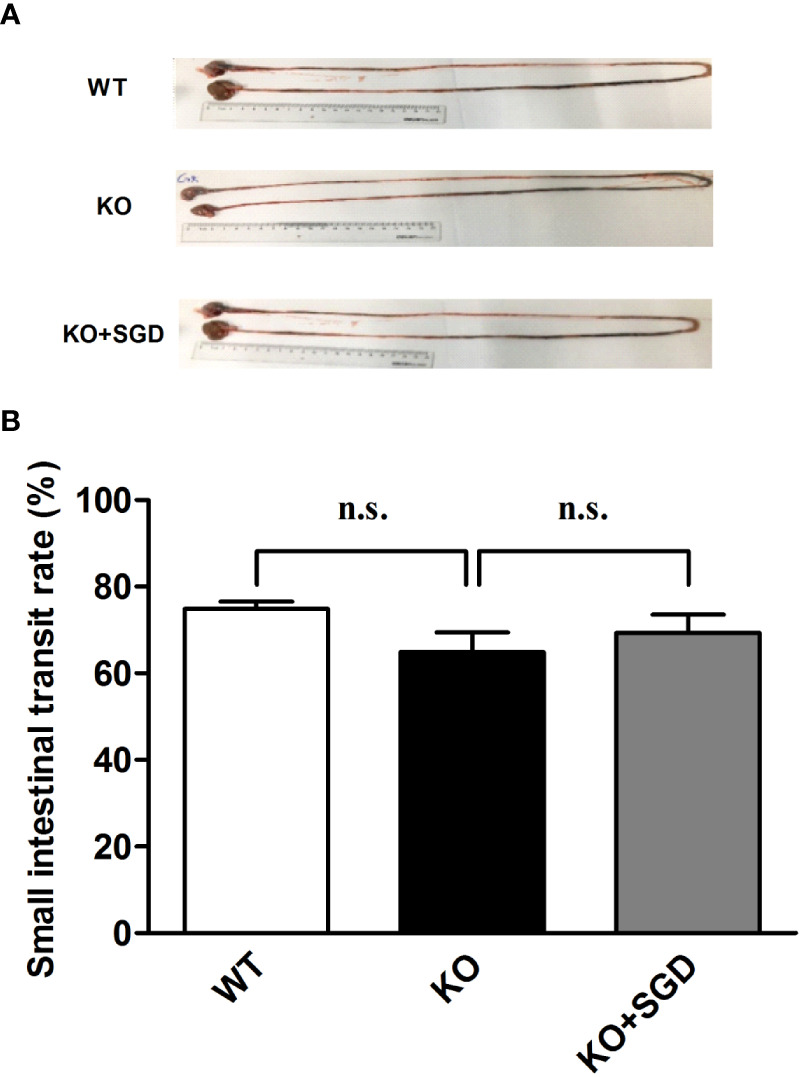
Small-intestinal charcoal propulsion rate in rats and the effect of SGD. **(A)** Small-intestinal charcoal propulsion diagram for rats in the three groups **(B)** Statistical graph of small-intestinal charcoal propulsion for rats in the three groups. All results expressed as mean ± SE (n = 6/group, n.s., no statistical significance).

We compared the small intestinal propulsion function of SERT-KO rats with that of WT rats *via* charcoal meal experiments. No significant difference in small intestinal charcoal propulsion rate was found between SERT-KO rats and WT rats (*P* > 0.05), indicating that *SERT* knockout had no significant effect on small intestinal propulsion function. There was also no significant difference in intestinal charcoal propulsion rates in SERT-KO rats gavaged with SGD or not (*P* > 0.05). This indicates that SGD did not affect the small intestinal propulsion function in SERT-KO rats (**Figure 3**).

### Spontaneous Contractile Activity of Ileal Smooth Muscle in SERT-KO Rats and the Effect of SGD

**Figure 4:**
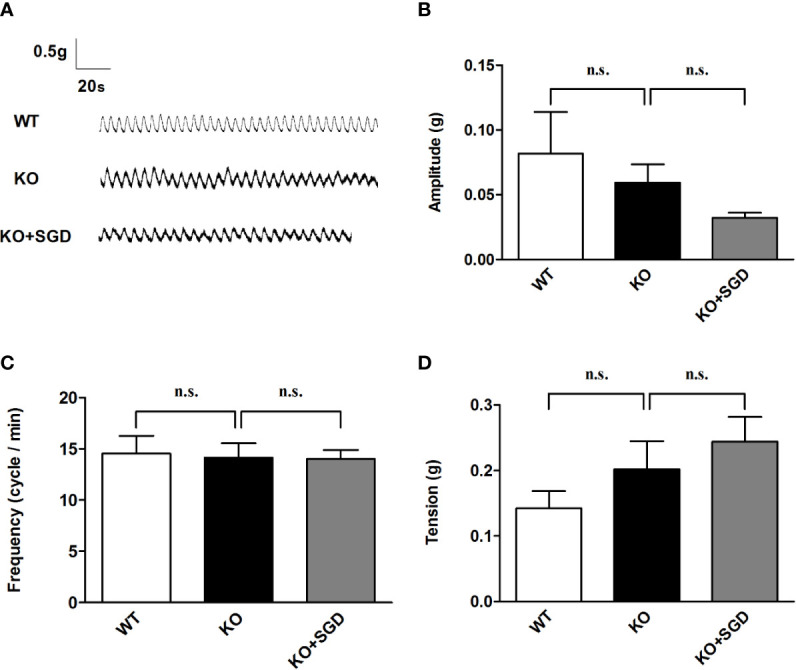
Spontaneous contractile activity of the ileal smooth muscle in rats and the effect of SGD. **(A)** Original recording traces **(B)** Summary data showing the average values of the amplitude. **(C)** Summary data showing the average values of the frequency **(D)** Summary data showing the average values of the tension of spontaneous contractile activity of the isolated ileal smooth muscle. All results are expressed as mean ± SE (n = 6/group, n.s., no statistical significance).

We assessed the spontaneous contractile activity of ileal longitudinal smooth muscle in SERT-KO and WT rats through organ bath experiments. It was found that the amplitude, frequency, and tension of spontaneous contraction of ileal longitudinal smooth muscle did not differ significantly between SERT-KO and WT rats (*P* > 0.05). SGD gavage did not elicit significant changes in the amplitude, frequency, and tension (*P* > 0.05) (**Figure 4**).

### ACh-Induced Contraction of Ileal Smooth Muscle in SERT-KO rats and the Effect of SGD

**Figure 5:**
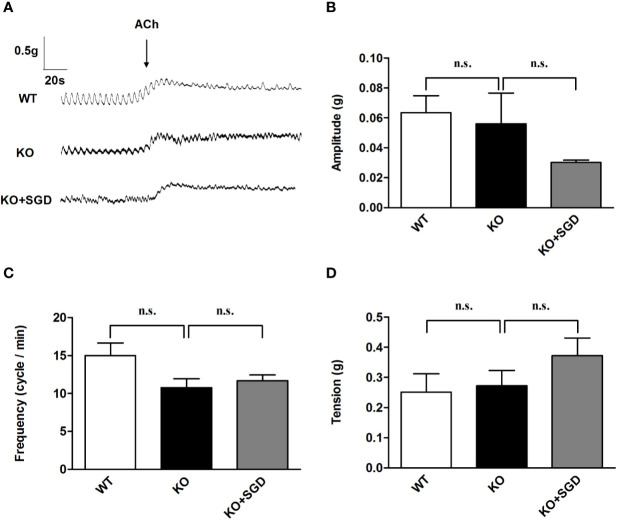
ACh-induced contraction of the ileal longitudinal smooth muscle in rats and the effect of SGD. **(A)** Original recording traces **(B)** Summary data showing the average values of the amplitude **(C)** Summary data showing the average values of the frequency **(D)** Summary data showing the average values of the tension of ACh-induced contraction of the isolated ileal smooth muscle. All results expressed as mean ± SE (n = 6/group, n.s., no statistical significance).

We assessed ACh-induced contraction of ileal longitudinal smooth muscle in SERT-KO rats and WT rats *in vitro*. It was found that there was no significant difference in the amplitude, frequency, and tension of ileal longitudinal smooth muscle contraction induced by ACh between SERT-KO and WT rats (*P* > 0.05). After SGD gavage in SERT-KO rats, the amplitude, frequency, and tension of isolated ileal smooth muscle contraction did not significantly change in the presence of ACh (*P* > 0.05) (**Figure 5**).

### KCl-Induced Contraction of Ileal Smooth Muscle in SERT-KO Rats and the Effect of SGD

**Figure 6:**
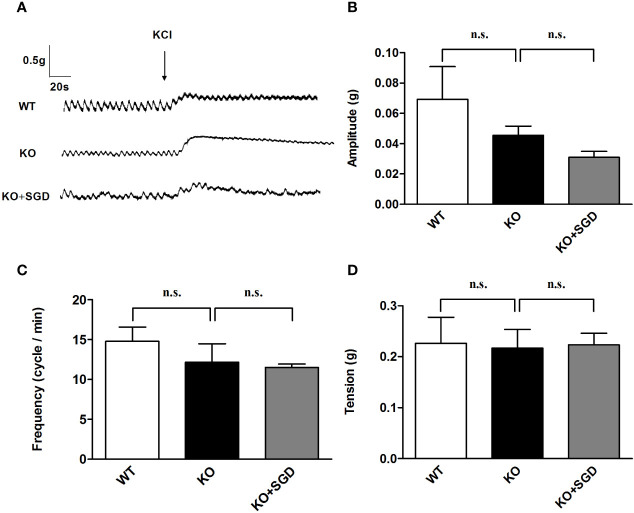
KCl-induced contraction of the ileal longitudinal smooth muscle in rats and the effect of SGD. **(A)** Original recording trace **(B)** Summary data showing the average values of the amplitude **(C)** Summary data showing the average values of the frequency **(D)** Summary data showing the average values of the tension of KCl-induced contractions of the isolated ileal smooth muscle. All results expressed as mean ± SE (n = 6/group, n.s., no statistical significance).

We assessed KCl-induced contraction of ileal longitudinal smooth muscle in SERT-KO rats and WT rats *in vitro*. There were no significant difference in the amplitude, frequency, and tension of ileal longitudinal smooth muscle contraction induced by KCl between SERT-KO and WT rats (*P* > 0.05). After SGD gavage in SERT-KO rats, the amplitude, frequency, and tension of contraction was not significantly changed in the presence of KCl (*P* > 0.05) (**Figure 6**).

### Spontaneous Contractile Activity of the Colonic Smooth Muscle in SERT-KO Rats and the Effect of SGD

**Figure 7:**
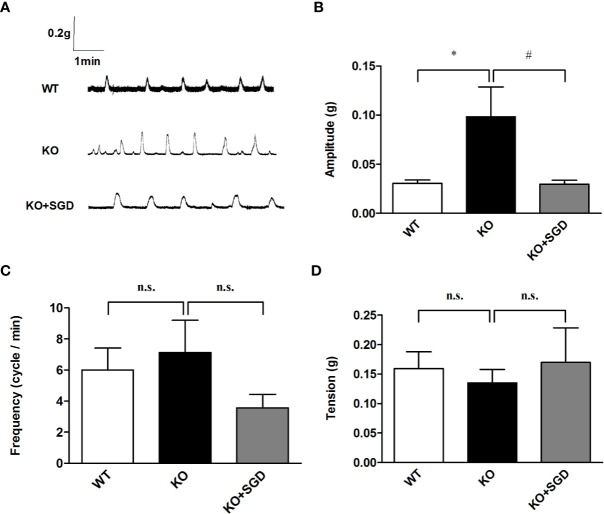
Spontaneous contractile activity of the colonic smooth muscle in rats and the effect of SGD. **(A)** Original recording traces **(B)** Summary data showing the average values of the amplitude **(C)** Summary data showing the average values of the frequency **(D)** Summary data showing the average values of the tension of spontaneous contractile activity of the isolated colonic smooth muscle. All results are expressed as mean ± SE (n = 6/group, *P < 0.05 vs. WT, ^#^P < 0.05 vs. KO, n.s., no statistical significance).

We observed the spontaneous contractions of the colonic longitudinal smooth muscle in SERT-KO rats and WT rats *in vitro*. It was found that compared with that in WT rats, there was no statistical difference in the frequency and the tension of spontaneous contraction of the colonic longitudinal smooth muscle in SERT-KO rats (*P* > 0.05), but the amplitude was significantly increased. (*P* < 0.05). After the intervention of SGD in SERT-KO rats, there was no significant changes in the frequency and the tension of spontaneous contraction of the colonic longitudinal smooth muscle (*P* > 0.05), but the amplitude significantly decreased (*P* < 0.05) (**Figure 7**).

### ACh Induced Contraction of the Colonic Smooth Muscle in SERT-KO Rats and the effect of SGD

**Figure 8:**
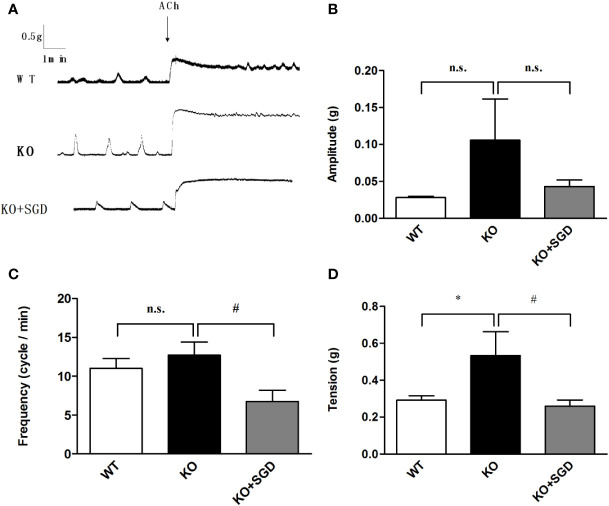
ACh-induced contraction of the colonic smooth muscle in rats and the effect of SGD. **(A)** Original recording traces **(B)** Summary data showing the average values of the amplitude **(C)**. Summary data showing the average values of the frequency **(D)**. Summary data showing the average values of the tension of spontaneous contractile activity of the isolated colonic smooth muscle. All results are expressed as mean ± SE (n = 6/group, *P < 0.05 *vs.* WT, ^#^ *P* < 0.05 *vs.* KO, n.s., no statistical significance).

We assessed ACh-induced contractile activity of the colonic longitudinal smooth muscle in SERT-KO rats and WT rats *in vitro*. Although there were no significant differences in the amplitude and the frequency of the colonic longitudinal smooth muscle contraction induced by ACh between SERT-KO rats and WT rats, the tension significantly increased (*P* > 0.05). After SGD gavage in SERT-KO rats, the frequency and amplitude but not the tension of isolated colonic smooth muscle contraction significantly decreased in the presence of ACh (*P* < 0.05) (**Figure 8**).

### KCl-Induced Contraction of the Colonic Smooth Muscle in SERT-KO Rats and the Effect of SGD

**Figure 9:**
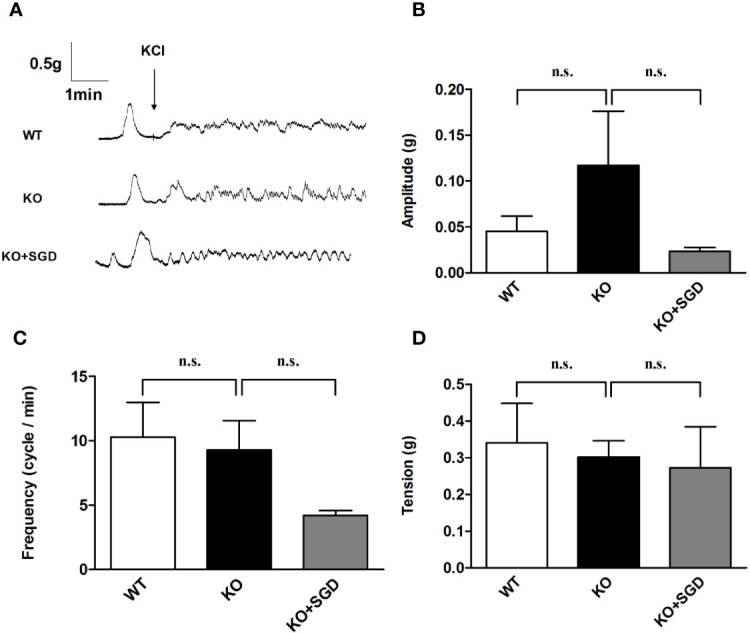
KCl-induced contraction of the colonic smooth muscle in rats and the effect of SGD. **(A)** Original recording traces **(B)** Summary data showing the average values of the amplitude **(C)** Summary data showing the average values of the frequency **(D)** Summary data showing the average values of the tension of spontaneous contractile activity of the isolated colonic smooth muscle. All results expressed as mean ± SE (n = 6/group, n.s., no statistical significance).

We assessed KCl-induced contractile activity of the colonic longitudinal smooth muscle in SERT-KO rats and WT rats *in vitro*, and no significant differences were found in the amplitude, frequency, and tension of the colonic longitudinal smooth muscle contraction induced by KCl (*P* > 0.05). After SGD gavage in SERT-KO rats, the amplitude, frequency, and tension of contraction did not significantly change in the presence of KCl (*P* > 0.05) (**Figure 9**).

### Expression of Muscarinic Receptor Protein in Colon Tissues of SERT-KO Rats and the Effect of SGD

**Figure 10:**
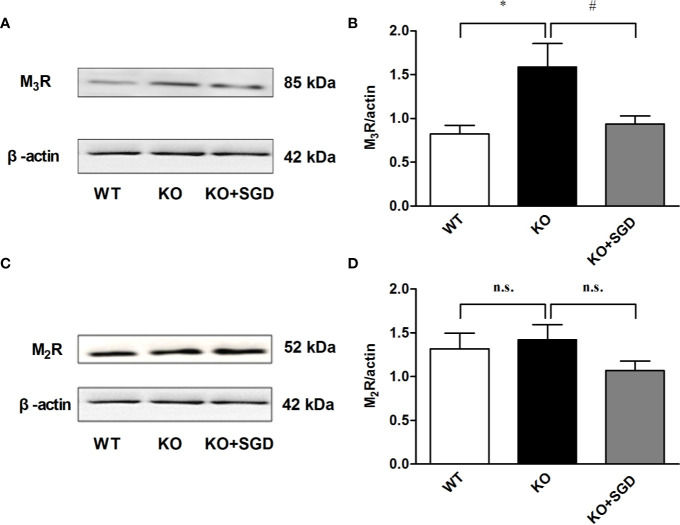
The expression of muscarinic receptor protein in colon tissues of SERT-KO rats and the effect of SGD. **(A)** The band diagram of M_3_R expression **(B)** Summary data showing the average ratio of M_3_R band gray value to β–actin band gray value **(C)** The band diagram of M_2_R expression **(D)** Summary data showing the average ratio of M_2_R band gray value to β–actin band gray value. All results are expressed as mean ± SE (n = 6/group, *P < 0.05 *vs.* WT, ^#^ *P* < 0.05 *vs.* KO, n.s., no statistical significance).

Western blotting was performed to assess the expression levels of M_3_R and M_2_R in the colon tissues of SERT-KO and WT rats, and M_3_R expression in SERT-KO rats was found to be significantly up-regulated (*P* < 0.05). After SGD gavage, M_3_R expression of SERT-KO rats was significantly down-regulated (*P* < 0.05) (**Figure 10**).

## Discussion

Gastrointestinal motility is critical for normal intestinal physiological function. Gut motor disorder is one of the primary pathophysiological changes underlying IBS. Unregulated intestinal motility occurs in 25% to 75% of patients with IBS. The symptoms of IBS, such as lower abdominal pain and abnormal defecation, are closely related to disordered intestinal motility. Compared with that in healthy individuals, increased colonic contractions are reported in IBS-C patients and decreased colonic contractions in IBS-D patients. Segmental activity of the sigmoid colon decreased in patients with IBS-D, but increased in patients with IBS-C (Clemens et al., 2003). Experimental studies have shown that colon supernatant from patients with IBS can weaken spontaneous and ACh-induced contraction of the isolated colonic smooth muscle, independent of IBS subtype (Guarino et al., 2017). In addition to that of the colon, patients with IBS-D also experience dysfunction of small intestinal movement (Kellow et al., 1999). Small-bowel transit time has been reported as normal or rapid in patients with IBS-D (Kellow et al., 1999). Clinical studies show that migrating motor complex (MMCs) in IBS patients is shorter than that in normal patients (Kellow and Phillips, 1987).

5-HT is widely distributed in the central and peripheral nervous system. It plays an important role in regulating gastrointestinal motility and sensory function. In the intestine, 90%- 95% of 5-HT is released by the enterochromaffin cells (ECs) (Gershon, 2004; Gershon and Tack, 2007). 5-HT exerts its function by activating the 5-HT receptor family. Abnormal 5-HT levels are considered an important factor in the pathogenesis of IBS. Some studies show that the diarrhea and the enhancement of gastrointestinal movement may be related to increased 5-HT signaling. The most consistent findings are the increased 5-HT levels in plasma in diarrheal diseases such as IBS-D, and decreased 5-HT plasma levels in individuals with constipation (Bearcroft et al., 1998; Gershon, 2013). In animal experiments, the loss of the intestinal neuron system and 5-HT can lead to abnormal gastrointestinal motility in adult animals (Li et al., 2011). IBS model rats subjected to combined chronic and acute bondage stress had lower hippocampal levels of 5-HT and increased 5-HT expression in the ileum and colon tissues (Yu et al., 2019). In IBS model rats stimulated by chronic water avoidance stress, the content of 5-HT in the colon tissues also increased significantly, whereas that in the hippocampus decreased significantly (Yu et al., 2014).

SERT abrogates 5-HT activity *via* reuptake from interstitial spaces. Studies have shown that the intestinal function of SERT-KO mice was abnormal; this manifested as increased water content in the feces and increased contraction in the distal colon (Chen et al., 2001). A clinical study reported down-regulated protein expression of SERT in both adults and children with IBS-D (Faure et al., 2010).

To examine the effect of SERT on gut motor function in IBS, we assessed intestinal motility in SERT-KO rats. In our experiment, comparing the defecation of SERT-KO rats with that of WT rats, it was found that the former was significantly increased, indicating that intestinal peristalsis was accelerated in the SERT-KO rats. However, no alteration in small intestinal propulsion was found, suggesting that the changes in gut motor function occurred mainly in the colon.

To further investigate the mechanism underlying this phenomenon, we observed the muscle contraction function of the small intestine and colon in both SERT-KO rats and WT rats through organ bath experiments. Our results showed that spontaneous contraction of the ileal longitudinal smooth muscle was comparable between SERT-KO rats and WT rats, as were the responses to KCl and ACh. In regard to colonic motor function, we found that the amplitude of colonic spontaneous contraction was significantly increased in SERT-KO rats, and that this was repressed by SGD. The tension due to ACh-induced colonic muscle contraction in SERT-KO rats was higher than in WT rats; this was decreased by SGD.

ACh is an endogenous neurotransmitter released by cholinergic neurons in the enteric nervous system (ENS). The main source of ACh in the gastrointestinal tract is a network of myenteric neurons that control peristalsis and muscle tone through the activation of metabotropic M_2_ and M_3_-type muscarinic receptors (Gallegos-Perez et al., 2014). The spontaneous contraction of colonic longitudinal smooth muscles and their response to ACh was significantly increased in the model rats under maternal and infant separation combined with bondage stress (Jia et al., 2018). To investigate the molecular mechanism of muscle contraction dysfunction in SERT-KO rats in detail, we examined the expression of the muscarinic receptors, including M_3_R and M_2_R, in colon tissues. The expression of M_3_R, but not that of M_2_R, in SERT-KO rats was obviously higher than in WT rats. After SERT-KO rats were gavaged with SGD, M_3_R expression significantly decreased, suggesting that up-regulated M_3_R expression is involved in colonic dysmotility in SERT-KO rats, which can be regulated by SGD.

In summary, our data indicate that SERT-KO rats are a potential animal model of IBS because they manifest IBS-like gut motor dysfunction. A potential limitation of the present study is the sample size, and additional experiments are needed to verify our findings and explore further mechanisms underlying the alteration of gut motor function in SERT-KO rats. In addition, animal models cannot fully reflect the characteristics and pathophysiology of human disease, and the role of M_3_R in the pathogenesis of IBS warrants investigation in IBS patients. As an experiential prescription, the therapeutic effects of SGD on IBS have been proven in clinical practice. Its mechanisms of action remain poorly understood, and further studies are needed to elucidate these. Moreover, we will investigate the synergistic effect of the principal ingredients of SGD in the future.

## Data Availability Statement

All data of this study are included in this article.

## Author Contributions

YW, EW, YM, and ZB carried out the animal experiments and wrote the paper. YD analyzed the data. SS performed the HPLC analysis. CZ and HF monitored the reproduction and feeding of SERT-knockout rats. JY designed the experiments and revised the paper. All authors contributed to the article and approved the submitted version.

## Funding

This work was supported by grants from the National Natural Science Foundation of China (No. 81473630) (No. 81874391), Xinglin Scholar, and Xinglin Young Talent Program (No. RC-2017-02-07).

## Conflict of Interest

The authors declare that the research was conducted in the absence of any commercial or financial relationships that could be construed as a potential conflict of interest.
